# Exposure to pistachio pesticides and stillbirth: a case-control study

**DOI:** 10.4178/epih.e2016016

**Published:** 2016-04-30

**Authors:** Saeid Razi, Mohsen Rezaeian, Fatemeh Ghani Dehkordi, Azita Manshoori, Reza Goujani, Reza Vazirinejad

**Affiliations:** 1 Department of Epidemiology, School of Medicine, Rafsanjan University of Medical Sciences, Rafsanjan, Iran; 2 Department of Social Medicine and Environmental Research Center, School of Medicine, Rafsanjan University of Medical Sciences, Rafsanjan, Iran; 3 Department of Operation Room, School of Paramedical Sciences, Bushehr University of Medical Sciences, Bushehr, Iran; 4 Department of Gynecology, School of Medicine, Rafsanjan University of Medical Sciences, Rafsanjan, Iran; 5 Social Determinants of Health Research Center, Social Medicine Department, School of Medicine, Rafsanjan University of Medical Sciences, Rafsanjan, Iran

**Keywords:** Stillbirth, Pregnancy, Pesticides, Case-control studies, Iran

## Abstract

**OBJECTIVES:**

Stillbirth is an undesirable outcome of pregnancy. In light of the increasing use of pesticides and growing concerns about the possible health effects of agricultural pesticides, we investigated the effect of exposure to pistachio pesticides on stillbirth in pregnant mothers.

**METHODS:**

This case-control study was conducted in Rafsanjan, Iran from 2011 to 2012. A total of 125 females who had a recent stillbirth were included as the case group, and 250 controls were selected from females who had a recent live birth. For each case, two controls with the nearest propensity score to the case were selected. Data were collected using a protocol developed by the researcher that involved interviewing respondents and reviewing their medical records. Conditional multivariate and univariate logistic regression analysis were performed and odds ratios (ORs) and 95% confidence intervals (CIs) were calculated.

**RESULTS:**

The ORs of stillbirth in mothers living in pistachio gardens and those who were exposed to sprayed pesticides, in comparison to the controls, were 14.1 (95% CI, 3.3 to 63.4) and 5.0 (95% CI, 1.2 to 28.6), respectively. No significant differences were found in stillbirth rates according to the distance between the mother’s residence and a pistachio garden or involvement in agricultural activities.

**CONCLUSIONS:**

The results of our study showed that exposure to pistachio pesticides during pregnancy may increase the likelihood of stillbirth in mothers.

## INTRODUCTION

Stillbirth is an undesired outcome of pregnancy, and is defined as fetal death occurring after 20 weeks to 22 weeks of pregnancy or the birth of a fetus weighing 500 g that has died prior to or during childbirth [1]. The rate of stillbirth varies depending on geographic and socioeconomic factors [2]. The stillbirth rate has been reported to be 5.3 and 25 per 1,000 births in developed and developing countries, respectively [3]. Based on the literature, 97% of cases of stillbirth are seen in developing countries [4]. The rate of stillbirth was found to be 6.4 per 1,000 births in a study conducted in a province of Iran [5]. Stillbirth is recognized as a phenomenon imposing considerable economic and emotional impacts on both the affected families and society [6].

The factors related to stillbirth have not all been identified, although previous studies have characterized fetal, maternal, and placental factors [7]. The fetal factors, encompassing 25% to 40% of the cases, include structural abnormalities, aneuploidy, ascending infection from the water bag, and some infectious factors, such as rubella and toxoplasmosis. The placental elements include abruption placentae, infections, and placental infarcts. The most important maternal factors are diabetes, thrombophilia, abnormal delivery, infections, thyroid disease, high body mass index (BMI), smoking, and exposure to pesticides [7-9].

Exposure to pesticides is one of the most important environmental factors that increase the risk of stillbirth and birth defects [9]. Pesticides are widely used to protect agricultural crops despite their known high risks for human health [10]. It should be noted that poisoning through pesticides is increasing in developing countries in comparison with developed countries due to higher rates of pesticide usage and the availability of pesticides [11]. Pesticides bring about short-term and long-term effects on human health. The short-term impacts include eye irritation, blisters, blindness, vomiting, dizziness, diarrhea, and death [12]. It is well-known that the long-term effects include cancer, neurological problems, immunological problems, and respiratory dysfunction, which can take place after months of exposure [13].

Stillbirth has been reported in a number of studies as a longterm repercussion of pesticide exposure in pregnant females [2,9,14]. Toxicological studies have demonstrated the sensitivity of the fetus to environmental exposure, and pesticide exposure three to eight weeks into pregnancy has the most adverse effects on the fetus [9]. Rafsanjan, Iran is one of the most important regions of pistachio production, with a total of 88,000 hectares of pistachio farms. Approximately 700 tons of pistachio pesticides are used annually in this county, 95% of which are organophosphorus pesticides. This study was conducted with the aim of determining the relationship between exposure to pistachio pesticides in pregnant females and the risk of stillbirth in Rafsanjan from 2011 to 2012.

## MATERIALS AND METHODS

This case-control study was conducted in Rafsanjan from 2011 to 2012. The Research Council of the Rafsanjan University of Medical Sciences approved the study. Rafsanjan is a county in Kerman Province in Iran. The statistical population of this study comprised all pregnancies that occurred in Rafsanjan during 2011 to 2012. The participants were Iranian mothers who had a medical file in Rafsanjan Nik-Nafs Maternity Hospital. Mothers of other nationalities (e.g., Afghan nationality) and mothers with a history of exposure to X-rays and/or other factors contributing to stillbirth, such as medication or trauma, were excluded from the study. Thus, two groups were selected from a total of 5,586 Iranian females who delivered in this hospital. The rate of stillbirth was 2.02 per 1,000 live births.

Cases were selected from females who had recently experienced stillbirth, and controls were selected from the females who had a recent live birth. A total of 125 females with a recent stillbirth were included as the case group, and 250 controls were selected from females who had a recent live birth. For each case, two controls with the nearest propensity score to the case were selected by using propensity score matching [15].

The propensity score was calculated using SPSS version 21.0 (IBM Corp., Armonk, NY, USA) by matching the following variables using a fuzzy matching scheme: maternal age, prior history of pregnancy (yes/no), smoking (yes/no), place of living (city/village), the quality of insurance (social-service providing/rural-based/other/not available), level of education (less than diploma, high school diploma, more than high school diploma), history of stillbirth (yes/no), employment (employed/homemaker), BMI (kg/m^2^), annual income, and gestational age (weeks) based on a fuzzy matching scheme. Data collection was carried out using a checklist of items that was developed according to the information in the written hospital records.

In this reserach paper, the two groups of the study were considered, and from each group, participants had the same variable were matched in order to remove the factors had affected on stillbirth.

Having estimated the propensity score for each mother, the first case was chosen randomly. Then, two controls with the closest propensity score were chosen as the controls for this case. In the next step, the second case was selected and two other mothers from the list of controls were selected based on their propensity scores. This procedure was continued until the last mother who experienced stillbirth was matched with the last two control mothers [15].

The data collection instrument included 20 items divided into two sections, including demographic information and items asking about pesticide exposure. This questionnaire was filled out by a trained expert using the patients’ medical files and based on interviews with mothers in the maternity hospital. In this study, exposure to pistachio pesticides during pregnancy was taken as the independent variable. A number of questions were used to measure this variable, including questions that asked about living in a pistachio garden, the distance between the mothers’ living place and a pistachio garden, taking part in farming activities, and personal experience with pesticide-spraying procedures. The dependent variable was the outcome of pregnancy, defined as either the occurrence or nonoccurrence of stillbirth. In order to collect the data, consent was obtained from both the case and control groups. The data were entered into R 3.2.0. revised (www.R-project.org/). In order to compare the quantitative variables between the case and control groups, the independent two-sample *t*-test was used. Additionally, the chisquare test and/or Fisher’s exact test was employed to compare the frequency distribution of the quantitative variables in both groups. All tests were two-sided. The relationship between exposure to pistachio pesticides and stillbirth was studied using a conditional logistic regression model after adjusting for confounding factors. The confounding factors included the sex of the child, gestational age (weeks), underlying diseases in the mother, medication, BMI (kg/m^2^), iron supplementation during pregnancy, the kind of delivery (normal vs. caesarean), the kind of pregnancy (wanted, unwanted), pregnancy care, and average salary. In the final conditional logistic regression models, the relationship between exposure to pistachio pesticides and stillbirth was reported as an odds ratio (OR), 95% confidence interval (CI), and *p*-value. The *p*-values <0.05 were considered to indicate statistical significance. Matching the groups and importing the data were performed using SPSS, while all statistical calculations were performed in R.

## RESULTS

The statistical tests indicated that the case and control groups were matched properly, based on variables including the mothers’ age at pregnancy, the mothers’ job, place of residence, and the type of insurance, smoking, and history of stillbirth (Table 1). The mean number of years of formal education was also similar in the two groups (10.9±3.7 and 11.4±3.0 for case and control, respectively; p=0.17).

The study results showed that mothers in the case group had a significantly shorter duration of gestational age than the control group (31.1±5.8 vs. 38.1±1.7 weeks, respectively; p<0.01). The BMI of mothers was 26.1±3.9 kg/m^2^ and 24.1±3.5 kg/m^2^ in the case and control groups, respectively (p<0.01). Caesarean sections were performed in 73.6% and 46.4% of the subjects in the case and control groups, respectively, which was found to be a statistically significant difference (p<0.01). No significant difference was found between the two groups in terms of sex of the child (55.2% and 56.0% of children were males in the case and control groups, respectively). The use of medication in pregnancy was reported by 28.8% (n=36) and 24.4% (n=61) of the mothers in the case and control groups, respectively, which was not a statistically significant difference (Table 2).

The results showed that 13 (10.4%) and two (0.8%) mothers had lived in pistachio gardens during their pregnancy in the case and control groups, respectively (p<0.01). Additionally, 15 (12%) and six (2.4%) mothers had experienced exposure to pesticides by spraying pistachio pesticides in the case and control groups, respectively (p<0.01) (Table 3).

The logistic regression model demonstrated that the OR of stillbirth for the mothers living in pistachio gardens during pregnancy was 14.1 (95% CI, 3.3 to 63.4) which was statistically significant (p<0.05). In addition, the OR of stillbirth in mothers involved in spraying pesticides was 5.0 (95% CI, 1.2 to 28.6) which was also statistically significant (p<0.05) (Table 4).

Although the distance from the place of residence to pistachio gardens in the case group was greater than the controls, the difference was not statistically significant (OR=1.3). The OR of stillbirth in mothers who took part in agricultural activities was not significant (OR=0.8) (Table 4).

## DISCUSSION

Our findings showed that the likelihood of stillbirth increased with exposure to pesticides, indicating that exposure factors such as living in a pistachio garden, the area of the mother’s residence nealy closed to the area where pesticides were used, and any experience with spraying procedures, would bring about higher rates of stillbirth in comparison with the control group. This finding corresponded to the results of a study that investigated the exposure to pesticides in males and females. However, the current study focused only on the effects of maternal exposure [16]. Additionally, another study found that the risk of stillbirth was higher in exposed females [17].

A previous study carried out in 1984 reported a significant relationship between maternal pesticide exposure and the OR of stillbirth. That study focused on the exact time of pesticide exposure during pregnancy and the duration of exposure; these factors were not taken into account in our study due to lack of access to such information. Pastore et al. [18] studied the distance between the mothers’ place of residence and pistachio gardens, finding a significant relationship with stillbirth, which is consistent with our study. Furthermore, a study focusing on mothers engaged in farming in Sudan found an increased probability of stillbirth in the pesticide-exposed mothers (farming or spraying procedures) [19].

A study of pesticide exposure according to the kind of pesticide and the month of pregnancy found a significant relationship between pesticide use and stillbirth. In that study, the month-to-month analysis showed a considerable in the third and fourth months of pregnancy. Additionally, the location of the mother’s residence within a one-mile distance of where pesticides were used showed a significant relationship with stillbirth [20].

In addition to pesticide exposure, a significant relationship was found between the duration of pregnancy and stillbirth in the current study, such that early pregnancy loss was predictable in the case group. Similar findings have also been reported by Yudkin et al. [21] and Cotzias et al. [22], indicating the presence of a significant relationship between the gestational age and stillbirth.

As mentioned, the sex ratio was similar among the two case and control groups in our study. This absence of any apparent relationship between the sex of the child and the risk of stillbirth agrees with the findings of the study conducted by Huang et al. [23]. It is worth noting that the factors involved in stillbirth include fetal growth impairment and the level of response of the mother to the fetus [24]. The better acceptance of the male in some communities and the higher support that mothers receive in pregnancies with a male fetus may lead to a decrease in side effects such as stillbirth.

Our results showed no statistical difference according to the distance from the mothers’ place of residence to a pistachio garden or agricultural activities during pregnancy. More investigation showed that our data for these two items may have been biased, as our respondents did not have a consistent definition of pistachio gardens, since a very old garden with no trees was still considered a pistachio garden. Therefore, the data regarding the distance between their place of residence and pistachio gardens were not accurate. Regarding agricultural activities, this item was problematic, as most people living in the study area consider themselves farmers, although they may help with pistachio farming for only a few days each year.

Regarding the other two variables—living in a pistachio garden during pregnancy and involvement with spraying pesticides —we found a significant effect on stillbirth among the mothers. These variables most likely had significant effects due to the fact that they estimated exposure directly, without requiring a nuanced understanding on the part of the participants.

In the current study, in addition to pesticide exposure, mothers in the case group had a significantly higher BMI. The average BMI in the case group fell into the overweight range, while it was in the normal range in the control group. Our results indicate that a higher BMI was associated with a greater risk of stillbirth, in accordance with the findings obtained by Kristensen et al. [25]. Since overweight and obesity could lead to chronic diseases such as hypertension and diabetes, it is probable that obese pregnant females are prone to stillbirth [26,27]. The effect of this variable was controlled for in this study, and exposure to pesticides nonetheless increased the risk of stillbirth.

Huang et al. [23] found a significant relationship between low income and stillbirth, a point that was also observed by Chibber [28]. The socioeconomic condition of people may affect their residence. Thus, mothers with a lower socioeconomic status may live in areas on the fringe of cities. Pistachio gardens are located mostly in these areas, such that living in such places increases the probability of stillbirth. In this study, efforts were made to match the two groups in terms of their socioeconomic condition, and the study results are therefore highly valid regarding the relationship of pesticides to stillbirth.

However, no significant relationship was found between pregnancy care and stillbirth, although Huang et al. [23] found a significant relation between lower medication use during pregnancy and stillbirth. McClure et al. [29] found that lower medication use led to an increase in stillbirths. In Iran, caring for pregnant females is a priority; in the current study, all pregnant females received medical care, and this variable had no confounding effect on the results.

The increased stillbirth risk in mothers exposed to pistachio pesticides compared to mothers in the control group shows that pesticides have undesired effects on pregnant mothers. Based on systematic programs to eliminate maternal exposure to pesticides, the risk of stillbirth among mothers has dramatically reduced in the area under investigation. We hope to take appropriate actions to further reduce the incidence of stillbirth.

The main limitation of this study was that measuring exposure and outcomes requires a reliable source of data. We used data recorded in the hospital as well as interviews. The quality and accuracy of our results depend primarily on the quality of the data, and we were unable to verify the accuracy of our data.

The retrospective nature of such data, their reflection of clinical goals, and the possibility of recall bias during the interviews are the main limitations of this study. In addition, lack of information on the exact time of exposure to pesticides during pregnancy, weather conditions, the direction of wind during spraying procedures, and the type of pesticides in question is another possible limitation.

## Figures and Tables

**Table 1.** Matched demographic characteristics of the case and control groups using the propensity score technique

Variables	Cases (n=125)	Controls (n=250)	p-value
Maternal age (mean±SD)	27.8±6.4	28.8±5.2	0.15
Place of residence			0.13
Urban	71 (56.8)	162 (64.8)	
Rural	54 (43.2)	88 (35.2)	
Job			0.77
Homemaker	120 (96.0)	242 (96.8)	
Employed	5 (4.0)	8(3.2)	
Smoking			0.78
Yes	4(3.2)	10 (4.0)	
No	121 (96.8)	240 (96.0)	
Insurance			0.90
Social-service provider	56 (44.8)	115 (46.0)	
Village-based insurance	41 (32.8)	74 (29.6)	
Others	23 (18.4)	52 (20.8)	
None	5 (4.0)	9 (3.6)	
History of stillbirth			0.66
Yes	5 (4.0)	6 (2.4)	
No	120 (96.0)	244 (97.6)	
Level of education			<0.01
Less than a diploma	44 (35.2)	78 (31.2)	
High-school diploma	58 (46.4)	128 (51.2)	
More than a high-school diploma	23 (18.4)	44 (17.6)	
First pregnancy			<0.01
Yes	49 (39.2)	97 (38.8)	
No	76 (60.8)	153 (61.2)	

Values are presented as number (%). SD, standard deviation.

**Table 2.** The frequency distribution of mothers in the case and control groups based on medical and health conditions

Variables	Cases (n=125)	Controls (n=250)	p-value
History of underlying disease			0.36
Yes	36 (28.8)	61 (24.4)	
No	89 (71.2)	189 (75.6)	
Iron supplementation during pregnancy			0.49
Yes	121 (96.8)	245 (98.0)	
No	4 (3.2)	5 (2.0)	
Kind of pregnancy			0.90
Wanted	113 (90.4)	227 (90.8)	
Unwanted	12 (9.6)	23(9.2)	
Pregnancy care			0.34
Yes	122 (97.6)	248 (99.2)	
No	3 (2.4)	2 (0.8)	
Sex			0.96
Female	56 (44.8)	110 (44.0)	
Male	69 (55.2)	140 (56.0)	
Gestational age (mean±standard deviation)	31.1±5.8	38.1±1.7	<0.01
Medication	36 (28.8)	61 (24.4)	0/36

Values are presented as number (%).

**Table 3.** Comparison of the variables relevant to exposure to pistachio pesticides in the case and control groups

Variables	Case (n=125)	Control (n=250)	p-value
Living in a pistachio garden			<0.01
Yes	13 (10.4)	2 (0.8)	
No	112 (89.6)	248 (99.2)	
Distance from place of residence to a pistachio garden (mile)			0.56
≤1	62 (49.6)	116 (46.4)	
>1	63 (50.4)	134 (53.6)	
Farming activities			0.88
Yes	51 (40.8)	104 (41.6)	
No	74 (59.2)	146 (58.4)	
History of pesticide-spraying activity			<0.01
Yes	15 (12.0)	6 (2.4)	
No	110 (88.0)	244 (97.6)	

Values are presented as number (%).

**Table 4.** The results of logistic regression analysis of the variables related to exposure to pistachio pesticides and stillbirth

Exposure variables	Univariable		Multivariable		Hosmer- Lemeshow goodness-of-fit test (p-value)	Area under the ROC curve
	OR (95% CI )	p-value	OR (95% CI)	p-value		
Living in a pistachio garden	13.2 (1.2, 82.1)	0.05	14.1 (3.3, 63.4)	0.04	0.80	0.92
Distance from residence to a pistachio garden (≤1 mile)	1.4 (0.4, 4.4)	0.56	1.3 (0.3, 6.7)	0.72	0.56	0.92
Agricultural activities	1.2 (0.4, 3.9)	0.76	0.8 (0.1,4.1)	0.75	0.76	0.92
Involvement in pesticide-spraying procedures	5.5 (2.1, 14.8)	0.05	5.0 (1.2, 28.6)	0.03	0.47	0.93

The logistic regression model controlled for the following variables: sex of the child (male, female), gestational age (weeks), underlying disease in the mother (yes/no), medication use during pregnancy (yes/no), body mass index, iron supplementation during pregnancy (yes/no), type of delivery (normal, caesarean), and kind of pregnancy (wanted, unwanted). OR, odds ratio; CI, confidence interval; ROC, receiver operating characteristic.
